# Deep Sedation In Patients Undergoing Atrioventricular Nodal Reentry Tachycardia Ablation

**DOI:** 10.5812/cardiovascmed.10719

**Published:** 2013-10-28

**Authors:** Amirfarjam Fazelifar, Ali Eskandari, Mohammadjafar Hashemi, Mostafa Alavi, Mohammadzia Totounchi, Azam Forghanian, Mahboubeh Zeighami, Zahra Emkanjoo, Majid Haghjoo

**Affiliations:** 1 Cardiac Electrophysiology Research Center, Rajaie Cardiovascular Medical and Research Center, Iran University of Medical Sciences, Tehran, IR Iran; 2 Department of Anesthesiology, Rajaie Cardiovascular Medical and Research Center, Iran University of Medical Sciences, Tehran, IR Iran; 3 Electrophysiology Laboratory, Rajaie Cardiovascular Medical and Research Center, Iran University of Medical Sciences, Tehran, IR Iran

**Keywords:** Atrio-Ventricular Nodal Reentrant Tachycardia, Deep Sedation, Ablation Techniques Introduction

## Abstract

**Background:**

General anesthesia and deep sedation can be used during cardiac EPS to relief pain and provide comfort and immobility, but many electrophysiologists avoid sedation for better arrhythmia induction.

**Objective:**

To determine anesthesia effects in ablation procedures in adults, we used intravenous anesthetic agents in patients who underwent slow pathway ablation.

**Patients and Methods:**

One hundred patients who were to undergo radiofrequency catheter ablation were randomly assigned to with and without intravenous anesthesia groups. All patients had palpitation with a documented electrocardiography (ECG) compatible with atrio-ventricular nodal reentrant tachycardia (AVNRT). We used propofol, fentanyl and midazolam for intravenous sedation. Electrophysiological parameters were checked for the two groups and compared before and after the ablation.

**Results:**

Electrophysiological parameters were not significantly different in the two groups. In the anesthetic group, patients were more satisfied with the procedure (P value < 0. 001).

**Conclusions:**

Intravenous anesthesia could be done safely in patients who underwent electrophysiological procedures. It had no effect on arrhythmia induction or slow pathway ablation in patients with documented AVNRT.

## 1. Background

Electrophysiological testing (EPS) and ablation are effective invasive procedures in symptomatic arrhythmic patients. Atrio-ventricular node reentrant tachycardia (AVNRT) is the most frequent arrhythmia in these patients. General anesthesia and deep sedation can be used during cardiac EPS to relief pain and provide comfort and immobility, but many electrophysiologists avoid sedation for better arrhythmia induction. The effect of anesthetic drugs on the conduction system are controversial and some reports have revealed associations between anesthetic drugs and supraventricular arrhythmia termination while others have demonstrate no effect from propofol and/or isoflurane on atrial/ventricular tissue or atrioventricular node function (1, 2). However, successful management of anesthesia in patients undergoing radiofrequency catheter ablation (RFCA) requires that the pathologic tachycardia remains inducible.

## 2. Objective

In this study we tried to evaluate the effect of anesthesia on AVNRT management.

## 3. Patients and Methods

A total of 100 consecutive patients were included during January to December 2011. All patients underwent radiofrequency catheter ablation (RFCA) for symptomatic arrhythmia compatible with AVNRT and were informed about the details of the procedure and the anesthesia protocol, if they were included in the anesthetic group. Written informed consent was obtained from all patients. All antiarrhythmic drugs had been discontinued for at least five half-lives before the procedure. All patients were categorized randomly into two groups of with (Group A) and without (Group B) anesthesia. Patients with double arrhythmias or other types of arrhythmias were excluded from the study.

### 3.1. Intravenous Anesthesia

An anesthesiologist was responsible for anesthesia induction and at least one anesthesia nurse was dedicated for vital signs monitoring (BP and pulse oximetry) and propofol administration. Anesthesia induction was performed with fentanyl (1 µ/kg), midazolam (0.03 mg/kg) and propofol (200µ/kg/min). When the patient could no longer be aroused, the infusion rate was decreased and adjusted according to the patient’s condition. An oral airway was used for airway protection. A nasal canola was used with an O_2_ flow of 2 L/min. Cuff blood pressure monitoring and pulse oximetry were used for continuous vital signs monitoring. We did not use pre-medications such as atropin because of electrophysiological effects. All patients recovered within 30 minutes after anesthesia protocol termination. 

### 3.2. Electrophysiological Study

In the anesthetic group (Group A) catheter placement was performed after anesthesia induction and there was no response to the painful stimulation. In the non-anesthetic group (Group B), catheter placement was done after local anesthesia. All patients received four mapping catheters in the right atrium, right ventricular apex, His bundle region and coronary sinus. Intracardiac electrogram was recorded and displayed on a computerized multichannel recording system (Bard EP). Electrophysiological tests including atrial, coronary sinus and ventricular pacing were performed for arrhythmia induction. If no arrhythmia was inducible, isoproterenol infusion was administered to increase baseline heart rate by more than 25% and electrophysiological tests were done again for arrhythmia induction. We could induce at least two echo beats in all patients with or without isoproterenol infusion before the ablation. 

## 4. Results

There were 61 females and 33 males at the end of follow ups (mean age: 46 ± 13;range 8 to 77). Table 1 shows the characteristics of patients in the two groups. No anesthesia complication, except transient oxygen desaturation, was seen during the procedure. Electrophysiological characteristics are summarized in Table 2. After successful slow pathway ablation or modification, no more than one echo beat was induced in the patients with and without isoproterenol infusion up to three extra-stimuli from right atrium and coronary sinus. We could not induce arrhythmias from the right ventricle for patients in whom tacharrhymias were inducible from the right ventricular apex before ablation. Post ablation electrophysiological characteristics are summarized in Table 3. All patients stayed in the hospital for rhythm and hemodynamic monitoring for 24 to 48 hours. The patients mean clinical follow up was 6 months (Table 3). Fifty patients enrolled in each group to compare the effect of anesthesia on AVNRT induction and ablation but only 49 patients in the anesthetic group and 45 patients in the control group completed the entire follow up period. Only one clinical recurrence of tachyarrhythmias was suspected in each group (P value = 0.327). Before discharge, we asked patients about their satisfaction of the procedure. We asked them to give a score from very low to high (low, average, good and very good). In the anesthetic group patient satisfaction was significantly better than the other group (P value < 0.0001) (Table 4). 

## 5. Discussion

This study demonstrated that intravenous anesthesia is a safe and effective procedure for patients undergoing slow pathway ablation. No significant sinus pause, sinus bradycardia or Atrioventricular (AV) block was seen during anesthesia. Lai et al. showed that intravenous propofol has no brady-arrhythmic effects (3). Table 2 and 3 demonstrate that, our anesthesia protocol has no effect on electrophysiological parameters. A few reports revealed that anesthetic agents have no significant clinical effects on sinoatrial node or Atrioventricular nodal (AVN) conductions (1, 3, 4). There are some reports concerning the non-inducibility of AVNRT after intravenous sedation (5), but these findings are restricted to pediatrics. We could induce at least two echo beats in all patients with documented supra ventricular tachyarrhythmia (SVT) compatible with AVNRT with or without anesthesia. Although anesthetic patients need more isoproterenol infusion for arrhythmia induction, but there was no significant difference between the two groups (Table 2). To the best of our knowledge, patient’s satisfaction after electrophysiological procedures was not checked in the previous articles (1-5). We suppose that the most important key is appropriate prematurity for arrhythmia induction. We used up to three extra-stimuli with minimum prematurity conducted from AV node with or without isoproterenol infusion in all patients for arrhythmia induction. All patients in the anesthetic group revealed their satisfaction after the procedure. On the basis of the current study, in adults, AVNRT induction is not related to anesthetic agents and it could be performed safely for all patients who undergo electrophysiological procedures for slow pathway ablation. Intravenous anesthesia increases patient’s satisfaction without increasing complication rate or inducing disturbance in electrophysiological parameters.

**Table 1.** Patients’ Characteristics with and without Anesthesia

	With Anesthesia (n = 49)	Without Anesthesia (n = 42)	P value
**Gender, female**	29 (59.2)	30 (71.4) ^b^	0.223
**Age, y**	47.24 ± 14.33	46.12 ± 12.37 ^c^	0.692
**Weight**	75.16 ± 14.56	75.48 ± 14.74	0.919
**LVEF** ^a^	54.49 ± 3.98	52.74 ± 8.42	0.198
**Known case of CAD**	2 (4.2)	1 (2.4)	> 0.99
**Known case of HTN**	7 (14.3)	6 (14.3)	> 0.99
**Known case of DM**	6 (12.2)	3 (7.1)	0.498

^a^ Abbreviations: CAD, coronary artery disease; DM, diabetes; HTN, hypertension; LVEF, left ventricular ejection fraction

^b^ The data are shown with No. (%)

^c^ The data are shown with Mean ± SD

**Table 2.** Pre-ablation Electrophysiological Characteristics of Patients with and without Anesthesia

	With Anesthesia (n = 49)	Without Anesthesia (n = 42)	P value
**SCL ** ^a^	763.23 ± 137.33 ^b^	730.12 ± 135.39	0.259
**PR interval ** ^d^	135.19 ± 31.26	135.33 ± 24.26	0.981
**QRS duration**	97.77 ± 50.79	91.17 ± 26.65	0.453
**QT interval**	396.09 ± 51.46	385.14 ± 45.26	0.292
**Sinus AH interval**	81.28 ± 20.87	86.00 ± 25.46	0.359
**Sinus HV interval**	50.33 ± 7.06	51.36 ± 11.05	0.620
**AVWP**	331.38 ± 56.86	315.00 ± 33.60	0.132
**VAWP**	323.07 ± 63.77	333.98 ± 72.29	0.462
**AERP-FP**	333.64 ± 76.56	299.19 ± 80.80	0.159
**AERP-AVN**	254.48 ± 64.80	253.64 ± 59.29	0.953
**Arrhythmia CL**	340.14 ± 54.70	346.97 ± 55.23	0.592
**Arrhythmia VA (HRA)**	67.53 ± 38.84	53.200 ± 20.80	0.054
**Pre-ablation Antegrade Jump**	22 (53.7) ^c^	17 (37.8)	0.139
**Pre-ablation Retrograde Jump**	2 (5.1)	2 (4.4)	0.883
**Pre-ablation Isoproterenol Usege**	9(22.5)	7 (15.2)	0.387

^a^ Abbreviations: AERP-FP, antegrade effective refractory period of fast pathway; AERP-AVN, antegrade effective refractory period of AV node; AVWP, atrio-ventricular wenckebach point; SCL, sinus cycle length; VAWP, ventriculo-atrial wenckebach point

^b^ The data are shown with Mean ± SD

^c^ The data are shown with No. (%)

^d^ All times are in millisecond

**Table 3.** Post-ablation Electrophysiological Characteristics of Patients with and without Anesthesia

	With Anesthesia ^a^ (n = 49)	without Anesthesia (n = 42)	P value
**SCL**	693.28 ± 211.65	648.17 ± 123.56	0.310
**AH interval**	105.25 ± 127.25	77.24 ± 18.95	0.245
**HV interval**	52.05 ± 12.98	51.31 ± 10.25	0.803
**AVWP**	396.28 ± 43.93	315.70 ± 43.90	0.265
**AERP-FP**	304.44 ± 45.85	290.00 ± 28.86	0.480
**AERP-AVN**	274.75 ± 57.92	268.96 ± 43.37	0.655
**Slow pathway ablation, No. (%)**	25 (25.2)	25 (61.0)	0.399
**Procedure time**	26.43 ± 22.50	32.15 ± 51.34	0.504
**Duration of Follow Up**	6.17 ± 3.31	6.39 ± 3.62	0.777

^a^ All the data are shown with Mean ± SD

Procedure time is in minutes, Duration of follow up is in months and other times are in millisecond. We analyzed slow pathway ablation vs. modification after AVNRT termination.

**Table 4.** Patient Satisfaction after AVNRT Ablation

	Very Low (n = 7)	Low (n = 28)	Intermediate (n = 7)	High (n = 37)	P value
**Patients without anesthesia, No. (%)**	7 (17.1)	27 (65.9)	6 (14.6)	1 (2.4)	0.0001
**With Anesthesia, No. (%)**	0 (0.0)	1 (2.1)	1 (2.1)	36 (76.6)	0.0001
